# The effect of time-restricted eating on arterial stiffness indices in men with metabolic syndrome: study protocol for a randomized controlled trial

**DOI:** 10.1186/s13063-024-08284-6

**Published:** 2024-07-22

**Authors:** Aliyeh Ghannadzadeh Yazdi, Mohammad Masoumvand, Elena Philippou, Alireza Hatami, Zahra Dehnavi, Hanieh Barghchi, Maryam Ahmadi-Khorram, Ali Jafarzadeh Esfehani, Mohsen Nematy

**Affiliations:** 1 Department of Nutrition, Faculty of Medicine, Mashhad University of Medical Sciences, Mashhad, 91779-48564 Iran; 2 Student Research Committee, Faculty of Medicine, Mashhad University of Medical Sciences, Mashhad, Iran; 3 Department of Life Sciences, School of Life and Health Sciences, University of Nicosia, Nicosia, Cyprus; 4 Department of Nutritional Sciences, King’s College London, London, UK; 5 Metabolic Syndrome Research Center, Mashhad University of Medical Sciences, Mashhad, 91779-48564 Iran

**Keywords:** Time-restricted eating, Intermittent fasting, Circadian rhythm, Pulse wave velocity, Metabolic syndrome, Arterial stiffness, Randomized controlled trial

## Abstract

**Background:**

Time-restricted eating (TRE) has been shown to be associated with improvements in some aspects of the metabolic syndrome. Nevertheless, only a few studies have addressed the effect of TRE on pulse wave velocity (PWV). We thus propose a randomized controlled trial to compare the effects of TRE with standard dietary advice on PWV and thereby present the protocol.

**Methods:**

Forty-eight participants will be assigned to either TRE or control groups using simple randomization. The TRE group will consume their meals during a 10-h period and experience 14 h of fasting. They will also be advised to consume their last meal no later than 20:00. Both groups will receive standard dietary advice. The participants will be followed for 6 weeks. The primary outcome will be changes in PWV. Laboratory measurements, including lipid profile, liver enzyme tests, fasting blood glucose (FBG), insulin concentrations, and insulin resistance, as well as anthropometric data, blood pressure, basal metabolic rate, appetite status, physical activity level, sleep quality, cognitive function, quality of life, and calorie intake, will be evaluated throughout the study.

**Discussion:**

The outcomes of this study will allow a comparison of the effects of TRE and standard dietary recommendations on PWV and other cardiometabolic factors in individuals with metabolic syndrome (MetS).

**Trial registration:**

Iranian Registry of Clinical Trials; code: IRCT20201230049889N1; registered on August 14, 2022. The registration of the trial is accessible at: https://www.IRCT.ir/trial/64485?revision=281341.

## Administrative information

**Table:** 

Note: the numbers in curly brackets in this protocol refer to SPIRIT checklist item numbers. The order of the items has been modified to group similar items (see http://www.equator-network.org/reporting-guidelines/spirit-2013-statement-defining-standard-protocol-items-for-clinical-trials/).	
Title {1}	The Effect of Time-Restricted Eating on Arterial Stiffness Indices in Men with Metabolic Syndrome: Study Protocol for a Randomized Controlled Trial
Trial registration {2a and 2b}.	Iranian Registry of Clinical Trials; Code: IRCT20201230049889N1; Registered on August 14, 2022. https://www.IRCT.ir/trial/64485?revision=281341. {2b} n/a; No items will be collected from the World Health Organization Trial Registration Data Set.
Protocol version {3}	This is the first version of the protocol, finalized on July 3, 2022.
This is the first version of the protocol, finalized on July 3, 2022.	The Vice Chancellery for Research and Technology of the Mashhad University of Medical Sciences will fund and provide the facilities and equipment required for the study [grant number: 4001494], without involvement in study design, collection, analysis, and interpretation of data, and in manuscript writing.
Author details {5a}	^1^ Department of Nutrition, Faculty of Medicine, Mashhad University of Medical Sciences, Mashhad, Iran ^2^ Student Research Committee, Faculty of Medicine, Mashhad University of Medical Sciences, Mashhad, Iran ^3^ Department of Life Sciences, School of Life and Health Sciences, University of Nicosia, Nicosia, Cyprus ^4^ Department of Nutritional Sciences, King’s College London, London, UK ^5^ Metabolic Syndrome Research Center, Mashhad University of Medical Sciences, Mashhad, Iran * Authors marked with an asterisk (*) have contributed equally as corresponding authors.
Name and contact information for the trial sponsor {5b}	n/a; This trial has no sponsor and is funded by the Vice Chancellery for Research and Technology of the Mashhad University of Medical Sciences.
Role of sponsor {5c}	n/a; This trial has no sponsor and is funded by Mashhad University of Medical Sciences.

## Introduction

### Background and rationale {6a}

Metabolic syndrome (MetS) is a set of simultaneous systemic abnormalities associated with increased risk for cardiovascular diseases (CVD) that was first introduced and discussed in the literature about three decades ago. Based on the International Diabetes Federation (IDF), MetS is defined as the presence of central obesity plus at least two other risk factors, including hyperglycemia, hypertriglyceridemia, low high-density lipoprotein (HDL), and hypertension [1]. Insulin resistance, which is believed to be the main mechanism driving MetS, increases the risk of type II diabetes mellitus (T2DM), CVD, and other diseases [2].

The global increase in the prevalence of MetS due to inactivity and obesity and its relationship with T2DM and CVD has become a subject of interest for researchers aiming to reduce the burden of MetS in both clinical and social settings [1, 2]. Scuteri et al. concluded that the pattern of MetS components differed between countries and cultures, and thus treatment approaches may need to also vary depending on the population [3].

The global prevalence of MetS is 20–25% [4, 5], while a meta-analysis of 69 Iranian studies over a period of 17 years reported that one third of Iranian adults had MetS. Prevalence varied by age and sex such that, in people under 40, males were prominently affected, whereas MetS prevalence was higher among postmenopausal women compared to age-matched men [6]. Furthermore, the patterns of MetS components differ between men and women [7]. Notably, MetS is more strongly associated with an increased risk for coronary heart disease (CHD) and CVD in women [8].

Nutrition, in terms of both quantity, quality, and timing of food intake, plays an important role in MetS development [9]. The suggested mechanism linking metabolic disease with meal irregularity is through effects on the circadian rhythm since food acts as a “zeitgeber,” i.e., “time keeper” [10]. The desynchronization of circadian rhythm due to irregular eating patterns causes lipid metabolism impairment, glucose intolerance, insulin insensitivity, and activation of inflammatory cascades [11]. Moreover, the insulin resistance resulting from meal irregularity activates several mechanisms associated with atherosclerosis. Insulin resistance leads to dyslipidemia, inflammation, and endothelial dysfunction, thereby increasing the risk of atherosclerosis [12]. In this regard, a link can be established between both meal irregularity and metabolic syndrome with arterial stiffness.

The effect of time-restricted eating (TRE) on MetS has been documented in several animal and human studies. It has been proposed that TRE can improve insulin insensitivity [13]. Furthermore, research has reported that TRE may lead to a reduction in body weight and blood pressure, as well as improvements in body composition, and lipid and glucose metabolic profiles [11]. TRE appears to be a safe intervention, with minimal adverse events reported in studies [14].

The cardiometabolic effects of TRE have also been investigated. A trial showed that a 12-week 10-h TRE significantly improved cardiometabolic indices in 12 individuals with MetS [15]. Zuo et al. conducted a study investigating the effects of a high-protein, intermittent fasting low-calorie (HP-IF-LC) diet on obese individuals. During the 12-week weight loss phase (first phase) of the study, they found a significant decrease in pulse wave velocity (PWV). In the 1-year weight maintenance phase (second phase) of the study, they compared a high-protein, intermittent fasting (HP-IF) diet with a heart healthy (HH) diet and observed a significant decrease in PWV [16]. A systematic review also found that Ramadan fasting, which involves restricting food and drink intake to a specific window between sunrise and sunset, improved lipid profile and anthropometric features in healthy individuals but not in diabetic individuals [17]. In another study, participants followed early time-restricted feeding (eTRF) with an eating window of 6 h compared to a 12-h eating period for 5 weeks. Eight overweight prediabetic males were assigned to intervention and control groups. The eTRF led to significant reductions in systolic and diastolic blood pressure, fasting insulin level, mean insulin level, and peak insulin value. They observed an improvement in PWV and augmentation index (AIx), although not statistically significant [13].

However, to the best of our knowledge, studies did not evaluate the effect of TRE alone on arterial stiffness, and some assessed the effects of TRE in populations other than individuals with MetS. A meta-analysis in 2020 suggested that despite the observed benefits of TRE in literature, randomized controlled trials (RCT) with larger sample sizes are necessary to further support the usefulness of TRE in prevention and treatment of MetS [11].

The proposed study aims to recruit a larger number of participants to assess the effect of a 6-week 10-h TRE protocol on PWV in individuals with MetS. Other cardiometabolic effects of TRE will also be investigated. It is hypothesized that TRE will reduce PWV, a marker of arterial stiffness, and improve the cardiometabolic profile in individuals with MetS. Considering the sex-related differences in MetS components and the potential effect of the menstrual cycle on metabolism, this study limits recruitment to men. With this approach, we will have a homogeneous group, which will reduce variability and minimize the confounding factors, leading to more robust results. Also, taking into account that the relationship between unhealthy lifestyles and MetS is stronger in men than in women [18], focusing this study on men may help develop gender-specific prevention and intervention programs.

## Objectives {7}

### Primary objectives

The primary objective is to determine the effect of a 10-h TRE compared to standard dietary recommendations on indices of arterial stiffness among men with MetS.

### Secondary objectives

The secondary objective is to determine the effect of 10-h TRE compared to standard dietary recommendations on:

- Anthropometric parameters and body composition outcomes assessed by bioelectrical impedance analysis (BIA)
- Energy metabolism outcomes assessed by indirect calorimetry (IC)
- Blood markers including lipid profile (triglyceride, total cholesterol, low-density lipoprotein (LDL), and high-density lipoprotein (HDL)), liver enzyme tests (aspartate aminotransferase (AST), alanine aminotransferase (ALT), and alkaline phosphatase (ALP)), fasting blood glucose (FBG), and insulin concentrations
- Insulin resistance assessed by the Homeostatic Model Assessment for Insulin Resistance (HOMA-IR) [19]
- Appetite status outcomes assessed by visual analog scale (VAS)
- Cognitive function outcomes assessed by the Go-No-Go (GNG) test and the Wisconsin Card Sorting Test (WCST)
- Quality of life outcomes assessed by the World Health Organization Quality of Life Brief Version questionnaire (WHOQOL-BREF*)*
- Sleep quality outcomes assessed by the Pittsburgh Sleep Quality Index (PSQI)
- Physical activity outcomes assessed by the International Physical Activity Questionnaire (IPAQ) and pedometer
- Calorie intake assessed by 3-day food and drink diary

## Trial design {8}

This study is designed as a randomized controlled trial (RCT), with parallel groups and an allocation ratio of 1:1, in a superiority design framework. Regarding the allocation of each participant to the control and intervention groups, randomization will be run using computerized random number generation and sealed envelopes will be used to conceal the random allocation. The potential participants will be identified among the population of the PERSIAN Cohort Study in Mashhad University of Medical Sciences and will be recruited through phone calls. Screening, including both physical and laboratory examinations, will be carried out to assess the presence of MetS based on IDF criteria [16]. The study design and planned assessments for each stage of the study are explained in detail, in the following sections, and briefly summarized in Table 1. The study protocol adheres to the checklist of the Standard Protocol Items: Recommendations for Interventional Trials (SPIRIT) guidance (Table 1).

**Table 1:** The timeline for the enrollment, intervention, and assessment schedule

	**Timepoint**	**Timeline**				
		**Enrollment**	**Allocation**	**Study period**		
				**Day 1**	**Week 3**	**Week 6**
	**Enrollment**	✔				
	**Eligibility screening**	✔				
	**Informed consent**		✔			
	**Allocation**		✔			
	**Intervention**					
	Intervention group: 10-h TRE					
	Control group: non-TRE					
**Fasting state assessments**	**Anthropometry**			✔	✔	✔
	**Bioelectrical impedance analysis**			✔	✔	✔
	**Pulse wave velocity**			✔	✔	✔
	**Indirect calorimetry**			✔		✔
	**Blood laboratory tests**					
	• Lipid profile: TG, TC, LDL, HDL			✔	✔	✔
	• Blood glucose			✔	✔	✔
	• Insulin concentration			✔	✔	✔
	• HOMA-IR			✔	✔	✔
	• CBC			✔		✔
	• hs-CRP			✔		✔
	• Liver enzymes: AST, ALT, ALP			✔		✔
	**Appetite VAS** (pre-prandial)			✔		✔
	**Breakfast (standard meal)**			✔		✔
**Post-prandial state assessments**	**Appetite VAS** (post-prandial)			✔		✔
	**Blood laboratory tests**					
	**1st hour post-prandial**					
	• Blood glucose			✔		✔
	• Insulin concentration			✔		✔
	**2nd hour post-prandial**					
	• Blood glucose			✔		✔
	• Insulin concentration			✔		✔
	• CBC			✔		✔
	• hs-CRP			✔		✔
	**Cognitive function tests**					
	Go-No-Go test			✔		✔
	Wisconsin Card Sorting Test			✔		✔
	**WHOQOL-BREF questionnaire**			✔		✔
	**PSQI questionnaire**			✔		✔
	**IPAQ**			✔		✔
	Pedometer*			a		b
	**Three-day food and drink diary**^**†**^			c	c	c

*Abbreviations*: *TRE*, time-restricted eating; *TG*, triglyceride; *TC*, total cholesterol; *LDL*, low-density lipoprotein; *HDL*, high-density lipoprotein; *HOMA-IR*, Homeostatic Model Assessment for Insulin Resistance; *CBC*, complete blood count; *hs-CRP*, high-sensitivity C-reactive protein; *AST*, aspartate aminotransferase; *ALT*, alanine aminotransferase; *ALP*, alkaline phosphatase; *VAS*, visual analog scale; *WHOQOL­BREF*, World Health Organization Quality of Life Brief Version; *PSQI*, Pittsburgh Sleep Quality Index; *IPAQ*, International Physical Activity Questionnaire

^*^Pedometer: daily evaluation, in the first 7 days and the last 7 days of the study period

^a^Throughout week 1

^b^Throughout week 6

^†^Three-day food and drink diary: daily evaluation

^c^Three consecutive days in weeks 1, 3, and 6

## Methods: participants, interventions, and outcomes

### Study setting {9}

This study will be conducted at the center of the PERSIAN Cohort Study at Mashhad University of Medical Sciences, at Imam Reza Academic Hospital, located in the North-East of Iran.

### Eligibility criteria {10}

#### Inclusion criteria

Participants will be men aged 18 to 65 years old, with documented MetS according to the IDF criteria [20] and a body mass index (BMI) < 35 kg/m^2^.

#### Exclusion criteria

Individuals who fall under the following categories will be excluded from the study: shift workers, individuals consuming food for less than 14 h per day, those with a documented diagnosis of diabetes mellitus, restrictive diets (e.g., cancer patients), thyroid disorders, eating disorders, receiving dietary supplements other than vitamins or minerals, and those unable to adhere to the TRE protocol.

### Who will take informed consent? {26a}

The principal researcher will inform eligible individuals about the study details, providing both written and verbal information in clear and comprehensible language. Subsequently, the interested individuals will be asked to provide written consent, in accordance with the standard procedures of the trial. Those who decline to give written consent or demonstrate significant distress will not be included in the study. In the case of illiterate individuals, since our study includes tests requiring literacy, it is not possible to recruit them.

### Additional consent provisions for collection and use of participant data and biological specimens {26b}

The consent form outlines the terms for data usage in case of participant withdrawal from the study and grants permission for sharing relevant data with specific academic experts or other regulatory authorities (ethics number: IR.MUMS.MEDICAL.REC.1401.213).

## Interventions

### Explanation for the choice of comparators {6b}

This study aims to evaluate the pure effects of TRE intervention on arterial stiffness indices. The comparison will be between the TRE group and the control group.

To mitigate the risk of contamination in TRE trials, where control group participants may adjust their eating times to mimic the intervention group, we emphasize the importance of maintaining their usual eating window and closely monitor their adherence to align with their designated group. Also, we planned to employ the appropriate statistical methods to assess and adjust the potential impact of contamination, as explained in the “Statistical methods” section.

### Intervention description {11a}

#### Intervention group (10-h TRE)

The participants will be instructed to limit all energy intake to a self-selected eating window of 10 h/day, with advice to consume the last meal no later than 20:00. Participants will choose the most suitable 10-h eating window with the researchers’ assistance. They will be free to consume non-energy-containing beverages during their fasting period. To avoid interfering with the participants’ sleep quality, they will be advised to avoid consuming tea and coffee after 20:00. Although no individualized instructions will be provided on dietary intake, participants will receive verbal recommendations and written healthy eating guidelines [21].

#### Control group (non-TRE)

The participants will not be instructed to limit their eating window. While no individualized dietary plans will be provided, participants will receive verbal recommendations and written healthy eating guidelines [21].

**Fig. 1:**
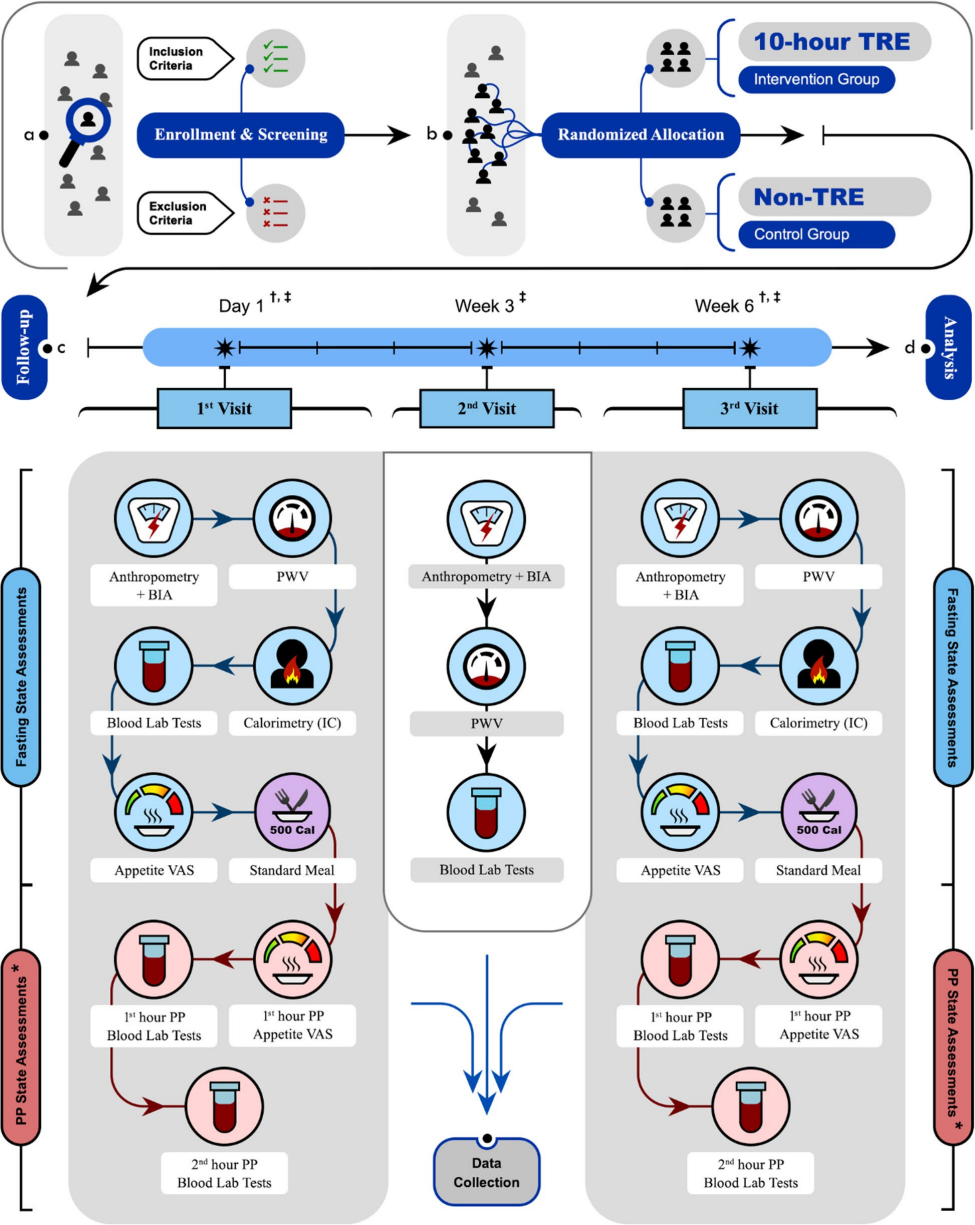
Summary of the study. “*” symbol indicates the following: the assessments of physical activity level (IPAQ), sleep quality (PSQI), cognitive function (GNG and WCST), and quality of life (WHOQOL-BREF) are assigned to this stage. “^†^” symbol indicates the following: *Pedometer* is used for daily physical activity evaluation, during the first 7 days and the last 7 days of the study period, throughout weeks 1 and 6. ^“‡^” symbol indicates the following: *Three-day food and drink diary* is used to record calorie intake for three consecutive days in weeks 1, 3, and 6. Abbreviations: *TRE*, time-restricted eating; *BIA*, bioelectrical impedance analysis; *PWV*, pulse-wave velocity; *IC*, indirect calorimetry; *VAS*, visual analog scale; *PP*, post-prandial

All participants will have a 45-min consultation with the study’s nutritionist on day 1 (baseline visit), week 3, and week 6 (final visit). Figure 1 provides a summary of the study protocol.

### Criteria for discontinuing or modifying allocated interventions {11b}

No specific side effects are anticipated following the TRE intervention. The potential reasons for discontinuing the intervention include the occurrence of any adverse effects due to the intervention or measurements or the participant’s unwillingness to continue participation. The Ethics Committee of Mashhad University of Medical Sciences (MUMS) will make decisions regarding the referred cases.

### Strategies to improve adherence to interventions {11c}

Compliance is defined as adhering to the planned 10-h TRE in the intervention group and avoiding eating time restrictions in the control group. Participants will be asked to write down the start and end times of food consumption in the prepared forms, which will allow us to improve adherence and identify any deviations from the prescribed eating window. Also, the participants will be asked to keep a food and drink diary including the time of consumption for 3 consecutive days at weeks 1, 3, and 6. Data collected from the reported 3-day food and drink diary will also be used in analyses. To further increase study compliance, a daily message will be sent to the participants in the TRE group to remind them of their eating window. Those participants who do not respond to 2 messages per week will be followed up by phone call.

### Relevant concomitant care permitted or prohibited during the trial {11d}

This trial allows all concomitant prescribed therapeutic interventions, in relevance to the measured parameters, without any restrictions. However, we will closely monitor for any changes in medications. If a participant needs to make a medication change, we will assess whether they should remain in the study on a case-by-case basis. Any change in medication dose will be considered a potential confounding factor. This approach will help to minimize confounding due to medication use and ensure the validity of our findings.

### Provisions for post-trial care {30}

A general practitioner and a nurse will be present at each visit to monitor the participants’ well-being during and at the end of the session. Following blood sampling, the participants will be assessed for any potential adverse incidents. Signs and symptoms, such as dizziness, puncture site bleeding, shortness of breath, blood pressure, and imbalance, will be carefully evaluated for any necessary intervention.

### Outcomes {12}

The primary outcome of this trial is to determine the effect of a 10-h TRE compared to standard dietary recommendations on indices of arterial stiffness among men with MetS. The secondary outcomes consist of changes in anthropometric parameters, indirect calorimetry parameters, lipid profile (triglyceride, total cholesterol, LDL, and HDL), liver enzyme tests (AST, ALT, and ALP), FBG and insulin concentrations, insulin resistance (assessed by HOMA-IR) [19], appetite status, cognitive outcomes (assessed by GNG test and WCST), quality of life assessment (WHOQOL-BREF questionnaire), sleep quality (PSQI questionnaire), physical activity assessments (IPAQ and pedometer), and 3-day food and drink diary.

### Participant timeline {13}

The schedule of enrolment, interventions, and assessments is shown in Table 1.

### Sample size {14}

The sample size was calculated according to the percentage of PWV change in the study by Sutton et al. [13]. Considering an alpha error of 5%, beta error of 20%, and an effect size of 0.63, a sample size of 20 was estimated for each of the two study groups.

$$n=\frac{{{\left({z}_{1-^{\alpha }\!\left/ \!_{2}\right.}+{z}_{1-\beta }\right)}^{2}(\sigma }_{1}^{2}+{\sigma }_{2}^{2})}{{\left({\mu }_{1}-{\mu }_{2}\right)}^{2}}$$

$$n=\frac{{\left({Z}_{1-^{\alpha }\!\left/ \!_{2}\right.}+{Z}_{1-\beta }\right)}^{2}}{{f}^{2}}$$

Taking into account a 20% loss to follow-up, 24 participants will be enrolled in each group.

### Recruitment {15}

The potential participants will be identified from the PERSIAN Cohort Study population at Mashhad University of Medical Sciences based on IDF criteria for MetS. They will be contacted via phone calls and invited to participate.

## Assignment of interventions: allocation

### Sequence generation {16a}

The recruited individuals will be allocated using a computer-generated sequence of random numbers, to the intervention and the control groups.

### Concealment mechanism {16b}

Each participant will receive a sealed envelope containing a random number which will assign them to either the intervention or control group.

### Implementation {16c}

An individual who is not part of the research team will be responsible for creating the allocation sequence, enrolling participants, and assigning them to the interventions.

## Assignment of interventions: blinding

### Who will be blinded {17a}

The type of intervention does not allow blinding for either participants or field researchers who will provide advice on the type of regimen. The statistical analysis however will be conducted by a blinded statistician, to minimize potential bias in the data analysis.

### Procedure for unblinding if needed {17b}

Considering that only the statistician will be blinded, unblinding would not be needed in any circumstances.

## Data collection and management

### Plans for assessment and collection of outcomes {18a}

Measurements will be conducted at baseline, week 3, and at the end of the intervention (week 6). All assessments will be conducted in the morning, following at least a 10-h fasting period. Before the visits, participants will be informed of the conditions required for the assessments and asked to comply with them.

#### Arterial compliance (at baseline, weeks 3 and 6)

The participants’ height will first be measured. The measurement will be taken in the morning following at least 6 h of fasting and abstinence from tobacco, alcohol, and caffeine consumption. The measurement will be taken in the supine position with their hands next to the body, following a 5-min rest at room temperature in a silent place [22].

The pulse wave analysis (PWA) will first be recorded, using a Sphygmocor (SphygmoCor Cardiovascular Management System software version 1.3; AtCor Medical Pty. Ltd., Sydney Australia) to assess the participant’s brachial systolic pressure, diastolic pressure, and pulse pressure. Three measurements will be taken and the average will be reported. Following this, aortic parameters including aortic systolic pressure, aortic diastolic pressure, aortic pulse pressure, mean arterial pressure (MAP), and heart rate (HR) will also be reported. Aortic augmented pressure, augmentation index (AIx), and AIx normalized for a heart rate of 75 beats/minute (AIx 75) will be calculated [22].

To measure pulse wave velocity (PWV), a femoral cuff will be fastened around the thigh, as high as possible. Three distances will be determined including the length from the femoral pulse sensation site to the top edge of the femoral cuff, the interval between the sternal notch to the femoral cuff, and the distance from the carotid pulse to the sternal notch in millimeters using a tape measure. The carotid pulse will be palpated and the tonometer will be positioned on it. When a regular carotid pulse is detected, the femoral cuff will be inflated. The detection of carotid waveforms will be continued until green waveforms are yielded. Then, the femoral cuff will deflate and the results will be recorded. The report will include heart rate, pulse wave velocity, and pulse transit time. Pulse transit time is the time taken for the pulse to travel from the carotid artery to the femoral artery, in milliseconds. PWV will be calculated by dividing the distance between the two arteries by the pulse transit time [22].

#### Anthropometric parameters (at baseline, weeks 3 and 6)

Weight will be measured in light clothing using electronic scales and height will be measured using a stadiometer. BMI will be calculated. Waist circumference will be measured at the midpoint between the lowest rib and the iliac crest. Bioelectrical impedance analysis (BIA; InBody 770; Smitech Pte Ltd; USA) will be undertaken to assess fat mass, muscle mass, and percent body fat.

#### Indirect calorimetry (at baseline and week 6)

To estimate energy requirements, the participant’s metabolic rate will be assessed during the fasting state (at least 6 h), using indirect calorimetry (IC). To initiate IC, the device (MetaLyzer 3B-R3 device; CORTEX Biophysik GmbH, Germany) will be calibrated. The participant will be asked to lie in the supine position and stay calm, immobile, and awake. The room will be quiet, at room temperature, and the participants should rest for at least 20 min. In case of any physical or psychological stress, the measurement will be postponed. In order to carry out IC, air samples (O2 and CO2) will be collected using a mask for a period of 20 min. Resting metabolic rate (RMR; kcal/d), diet-induced thermogenesis (DIT; kcal/d), substrate oxidation (SO; g/d), and respiratory quotient (RQ) will be measured.

#### Laboratory measurements (at baseline and week 6)

- *Fasting blood tests:* An 8-ml blood sample will be collected in the fasted state to assess complete blood cell count (CBC), liver enzyme tests (aspartate aminotransferase (AST), alanine aminotransferase (ALT), and alkaline phosphatase (ALP)), high-sensitivity C-reactive protein (hs-CRP), lipid profile (triglyceride, total cholesterol, low-density lipoprotein (LDL), and high-density lipoprotein (HDL)), fasting blood glucose (FBG), and insulin concentration. Insulin resistance and β cell function will be estimated using the Homeostatic Model Assessment for Insulin Resistance (HOMA-IR) calculator [23].
- *Postprandial assessment:* A standard breakfast test meal containing 500 kcal (50% carbohydrates, 20% protein, and 30% fat) will be provided at the beginning and end of the study. A 5-ml blood sample will be collected at 60 min following breakfast consumption, and blood glucose and insulin levels will be evaluated. Another 8 ml blood sample will be attained and CBC, hs-CRP, blood glucose, and insulin will be assessed at 120 min following the meal consumption.

#### Laboratory measurements (at week 3)

A 5-ml sample will be collected to assess the lipid profile (triglyceride, total cholesterol, LDL, and HDL), FBG, and insulin concentration. HOMA-IR will also be evaluated.

#### Appetite status (at baseline and week 6)

Appetite status will be assessed using visual analog scale (VAS) before breakfast and one hour after the meal. This scale comprises eight questions that address appetite and desire for specific food types. The test meal palatability will also be assessed using five other questions [24].

#### Physical activity assessment (at baseline and week 6)

Physical activity level will be assessed using a pedometer (Omron ri-Axis Pedometer- Step and Activity Tracker HJ-320; Kyoto, Japan) in the first and last 7 days of the study period. The participants’ activity will also be assessed using the International Physical Activity Questionnaire (IPAQ) at the beginning and end of the study. IPAQ includes 5 domains and assesses job-related physical activity, transportation physical activity, housework, house maintenance, and caring for family, recreation, sport, and leisure-time physical activity, and time spent sitting [25, 26]. The validated Persian version of IPAQ will be used [27].

#### Sleep assessment (at baseline and week 6)

The validated Persian version of the Pittsburgh Sleep Quality Index (PSQI) [28] will be used to assess sleep duration, time of sleep, and sleep quality at baseline and week 6. PSQI comprises 7 subscales including, subjective sleep quality, sleep latency, sleep duration, habitual sleep efficiency, sleep disturbances, use of sleeping medication, and daytime dysfunction. The total score ranges from 0 to 21, and participants with scores more than 5 will be considered to have poor-quality sleep [29].

#### Cognitive function assessment (at baseline and week 6)

Cognitive function will be evaluated using the Go-No-Go (GNG) test and the Wisconsin Card Sorting Test (WCST) [30, 31]. Both tests will be conducted using PEBL (Psychology Experiment Building Language), an open-source software developed for neuropsychological function experiments [32].

GNG is a simple experimental test that assesses inhibitory neurologic function. The participants will see a square consisting of four small squares arranged in a 2 × 2 structure. A letter P (Go signal) or R (No-Go signal) is hidden under a star in each of the four small squares. A star will be randomly faded, and the participants will be asked to press the right shift button whenever they see a P letter and not to press the button when they see an R letter. Before the start of the test, a short practice will be conducted to ensure that participants understand the instructions. No Go Errors and Reaction Time for Go Responses will be analyzed to interpret the results [33].

WCST is a card-matching test that evaluates neurocognitive flexibility. The participants will be presented with four category cards which are different in terms of colors, shapes, and number of shapes. A stimulus card will be shown to the participant. He should discover the rule through trial and error and sort the cards according to the rule. When the participant sorts the cards, feedback (correct or incorrect) will be shown on the screen after each sort. After six consecutive correct responses, the rule will be changed randomly and they have to identify the new rule [34].

WCST measures the total correct score, perseverative errors score, completed categories score, and failure to maintain a set score. The number of correct matches is regarded as the total correct score. The number of categories (color, shape, or number) that are learned correctly is defined as the completed categories score. The perseverative errors score means that the participant adhered to a previously correct rule, which is not correct anymore, despite showing incorrect feedback on the screen. The failure to maintain a set score is described as the number of making errors after fully learning the present rule [34].

#### Quality of life assessment (at baseline and week 6)

Quality of life will be assessed using the World Health Organization Quality of Life Brief Version (WHOQOL-BREF) questionnaire validated in Persian [35]. WHOQOL-BREF is a 26-item self-report scale that evaluates four domains of quality of life including physical, social, psychological, and environmental. Each item is scored one to five according to the Likert scale. The total score for each domain will be reported based on a 0 to 100 scale, and the higher the score, the better quality of life [36, 37].

#### Three-day food and drink diary (at week 1, 3 and 6)

To evaluate the individual’s calorie intake and compliance with the study protocol, participants will be asked to keep a detailed food and drink diary, at weeks 1, 3, and 6.

The subjects will be instructed to meticulously record every item they eat or drink, including the time of ingestion, portion sizes, cooking methods, and any additions like sauces. Participants will be required to document their intake for three consecutive days, including 1 day on a weekend. To ensure accuracy and prevent forgetting or underestimating their intake, participants will be asked to take quick notes using a paper diary. Throughout the study, researchers will be available to address any questions participants may encounter, review the diaries, and clarify any ambiguities or missing data during the follow-up visits.

### Expected outcomes

It is expected that arterial compliance will improve in the TRE group compared to the control group. In relation to secondary outcome measurements, an improvement in lipid profile, liver enzyme tests, FBG, insulin concentration, and insulin resistance is expected. There might also be improvements in cognitive outcomes, a reduction in the hs-CRP inflammatory marker, and changes in body weight and composition, with the latter possibly leading to small changes in RMR.

### Plans to promote participant retention and complete follow-up {18b}

Participants in the TRE group will receive daily messages to improve compliance, while all participants will be contacted and reminded of their scheduled visits.

### Data management {19}

In the current study, the database will be generated by entering data in the SPSS software version 20. To increase the accuracy of data collection, all data will be collected by the primary investigator. To ensure that the information collected during the study is kept securely and confidentially, each participant will be assigned a code that will be used to identify him throughout the study. Two separate, password-protected databases will be created, one containing the participants’ personal information (to which only the primary investigator will have access) and one containing the coded study data (to which the study team will have access). The written information, e.g., consent forms and any other data input forms will be kept in a locked cupboard in the primary investigator’s office. The final dataset will comprise all data collected on each participant identified using their unique code. Data sharing between investigators through networks will also be password-protected and access will be allowed only by investigators with a link sent by the primary investigator. All passwords will be changed every 3 months for security reasons.

### Confidentiality {27}

As explained in the “Data management” section, to ensure the confidentiality of data, each participant will be assigned a code throughout the study, and the databases will be password-protected. Accessing data will be allowed only by other investigators with a link sent by the primary investigator. All passwords will be changed every 3 months for security reasons. No participants will be identified in any publication or other dissemination activities and participants will be free to withdraw from the study without providing any reason and without their care by our team or other healthcare professionals being affected in any way. Collected data, of any kind, will be destroyed 5 years after completion of the study.

### Plans for collection, laboratory evaluation, and storage of biological specimens for genetic or molecular analysis in this trial/future use {33}

Blood samples will be collected from the median cubital vein at each visit. The samples will be centrifuged, and the resulting serum and plasma will be stored at – 80 °C until analysis [38]. Also, the remaining samples will be stored at − 80 °C, at Mashhad University of Medical Sciences for future use.

## Statistical methods

### Statistical methods for primary and secondary outcomes {20a}

The SPSS software version 20 will be used for data analysis based on the intention-to-treat method. Comparison between groups will be conducted by independent sample *t*-test and Mann-Whitney tests for quantitative data, while within-group comparisons will be assessed using paired sample *t*-test and Wilcoxon signed-rank tests. Comparison between groups will be conducted by chi-square test for qualitative data, and within-group comparisons will be assessed by McNemar’s test. Qualitative data (levels of physical activity based on IPAQ) will be presented using frequencies and percentages, while quantitative data (anthropometric parameters, arterial stiffness indices, indirect calorimetry parameters, laboratory tests, appetite VAS score, GNG score, WCST score, WHOQOL-BREF score, PSQI score, pedometer reports, 3-day food and drink diary reports) will be presented using mean and standard deviation or median and interquartile range depending on the data distribution. The relationship between outcomes will be assessed using Pearson’s correlation coefficient and Spearman’s rank correlation coefficient. A *p*-value of < 0.05 will be considered statistically significant.

To mitigate the risk of contamination in TRE trials, we will conduct sensitivity analyses to assess the impact of potential adjustments in the control group’s eating times. Furthermore, we will compare the results of the intention-to-treat analysis with per-protocol analyses, by checking the consistency of these results. Also, appropriate statistical methods will be employed to predict potential contamination and its impact on the study results.

### Interim analyses {21b}

This study will not include interim analyses. If frequent adverse effects are observed, the intervention will be halted, and a formal report will be submitted to the Ethics Committee of Mashhad University of Medical Sciences for their decision-making.

### Methods for additional analyses (e.g., subgroup analyses) {20b}

In this study, no additional analyses are intended to be conducted.

### Methods in analysis to handle protocol non-adherence and any statistical methods to handle missing data {20c}

The trial will use an intention-to-treat analysis protocol, to minimize the impact of participant dropouts and non-adherence to the protocol by analyzing all participants according to their original assignment, regardless of whether they completed the study or not. However, missing data from participants who drop out or do not adhere to the protocol will be imputed using statistical methods to ensure that the analysis will not be biased by missing data.

### Plans to give access to the full protocol, participant-level data, and statistical code {31c}

We are committed to promptly publishing the complete study protocol and findings. Upon reasonable request, the corresponding author is able to present an anonymized dataset and statistical code.

## Oversight and monitoring

### Composition of the coordinating center and trial steering committee {5d}

The Ethics Committee and Vice Chancellor of Research and Technology at Mashhad University of Medical Sciences will oversee and coordinate all phases of the study. These are academic units, free from any conflicting interests.

### Composition of the data monitoring committee, its role and reporting structure {21a}

The Ethics Committee of Mashhad University of Medical Sciences will be kept fully informed throughout the entire research process and will conduct at least two accuracy checks to ensure the integrity of the research.

### Adverse event reporting and harms {22}

TRE is a safe intervention, with only a few mild adverse events noted in some studies, according to a recent 2023 systematic review [14]. While severe adverse events are not expected in this study, minor events such as fatigue, dizziness, and headache may occur. These minor events will be monitored in terms of severity, time of occurrence, duration from the beginning of the intervention, and causality. This data will be reported to the Ethics Committee of Mashhad University of Medical Sciences for further decisions.

### Frequency and plans for auditing trial conduct {23}

The study protocol will undergo an assessment by a team of research ethics professionals, and the study procedure will be reviewed by the assigned supervisor after 5% of the study participants are recruited, in accordance with university regulations. At the end of the study, the research procedure will be evaluated, by the same assessment team.

### Plans for communicating important protocol amendments to relevant parties (e.g., trial participants, ethical committees) {25}

Any changes to the study’s protocol that may impact its conduct will be approved by the ethical committee of Mashhad University of Medical Sciences before being executed. Trial participants will be informed of the changes and their potential impact. The Iranian Registry of Clinical Trials will also be informed of any amendments conducted in the trial.

## Dissemination plans {31a}

Participants will receive a summary of their own results, upon the completion of the study. It will take approximately 4–6 months to prepare and disseminate the reports via journal publication. The reports will be shared irrespective of the results. The corresponding author will be accountable for all the conditions related to publishing the trial results, and there will be no limitations on publication imposed by the funder.

## Discussion

TRE and intermittent fasting (IF) are being investigated as approaches to improve cardiometabolic abnormalities in both animal and human trials [39–41] and have shown to be effective in various aspects of MetS [11, 15]. We planned a protocol to assess the effect of 10-h TRE on vascular stiffness in men with metabolic syndrome, aiming to contribute to the limited knowledge in this context.

We expect that TRE can efficiently improve arterial stiffness parameters, compared to the control group. The gold standard method of measurement for arterial stiffness is PWV, despite its dependence on age, blood pressure, and heart rate [42]. It can be proposed that the higher the PWV level, the higher the arterial stiffness [43]. The findings from this RCT will provide valuable insights into the potential benefits of TRE on arterial stiffness, which could inform future dietary guidelines and interventions for MetS patients. This study has several strengths including the presence of a control group, assessment of sleep quality as a confounding factor that may affect PWV [44], and assessment of different laboratory and clinical findings. By restricting the study to men and excluding those with a BMI ≥ 35 and comorbid conditions, the study population becomes more homogeneous, which may yield more reliable findings due to reduced confounding factors. The study may be limited in that participants’ diets may differ in terms of food quality. However, collecting detailed food and drink diaries at specific time points throughout the study will allow for assessing food composition. Also, participant recruitment and retention are significant practical issues in conducting this study, primarily due to the difficulties participants face in adhering to the TRE protocol. In this respect, the participants will be regularly followed up to assess study adherence, and a 20% rate of dropout was included in the sample size calculation to allow for attrition rate. Using an intention-to-treat analysis protocol also minimizes the effect of participant dropout.

In conclusion, this study is expected to lead to innovative findings regarding the effects of TRE on arterial compliance. Results from this and other studies can potentially lead to novel therapeutic approaches and help inform dietary guidelines on the timing of eating to improve MetS and cardiovascular outcomes with important individual and public health implications.

## Trial status

The trial was registered on ClinicalTrials.gov with the identifier IRCT20201230049889N1 on August 14, 2022. This is the first version of the protocol, which was finalized on July 3, 2022. The study is currently in the stage of participant recruitment, which has begun on October 23, 2023, and is expected to end approximately by the end of April 2024.

## Data Availability

Data sharing does not apply to this article, as no datasets are generated or analyzed yet so far, during the current study. But corresponding author may provide datasets upon reasonable request, after the collection and/or analysis of the datasets.

## Abbreviations

TRE: Time-restricted eating
PWV: Pulse wave velocity
FBG: Fasting blood glucose
MetS: Metabolic syndrome
CVD: Cardiovascular diseases
IDF: International Diabetes Federation
HDL: High-density lipoprotein
T2DM: Type II diabetes mellitus
eTRF: Early time-restricted feeding
AIx: Augmentation index
RCT: Randomized controlled trial
BIA: Bioelectrical impedance analysis
IC: Indirect calorimetry
LDL: Low-density lipoprotein
AST: Aspartate aminotransferase
ALT: Alanine aminotransferase
ALP: Alkaline phosphatase
HOMA-IR: Homeostatic Model Assessment for Insulin Resistance
VAS: Visual analog scale
GNG: Go-No-Go
WCST: Wisconsin Card Sorting Test
WHOQOL-BREF: World Health Organization Quality of Life Brief Version
PSQI: Pittsburgh Sleep Quality Index
IPAQ: International Physical Activity Questionnaire
SPIRIT: Standard Protocol Items: Recommendations for Interventional Trials
BMI: Body mass index
TG: Triglyceride
TC: Total cholesterol
CBC: Complete blood count
hs-CRP: High-sensitivity C-reactive protein
PP: Post-prandial
PWA: Pulse wave analysis
MAP: Mean arterial pressure
HR: Heart rate
AIx 75: AIx normalized for a heart rate of 75 beats/minute
RMR: Resting metabolic rate
DIT: Diet-induced thermogenesis
SO: Substrate oxidation
RQ: Respiratory quotient
IF: Intermittent fasting
